# Potent antitumour of the mTORC1/2 dual inhibitor AZD2014 in docetaxel‐sensitive and docetaxel‐resistant castration‐resistant prostate cancer cells

**DOI:** 10.1111/jcmm.16155

**Published:** 2021-01-28

**Authors:** Senmao Li, Jindong Sheng, Zhenhua Liu, Yu Fan, Cuijian Zhang, Tianjing Lv, Shuai Hu, Jie Jin, Wei Yu, Yi Song

**Affiliations:** ^1^ Department of Urology Beijing Key Laboratory of Urogenital Diseases (Male) Molecular Diagnosis and Treatment Center National Research Center for Genitourinary Oncology Peking University First Hospital and Institute of Urology Peking University Beijing China; ^2^ Department of Gynaecological Oncology Key Laboratory of Cancer Prevention and Therapy of Tianjin Tianjin’s Clinical Research Center for Cancer National Clinical Research Center for Cancer Tianjin Medical University Cancer Institute and Hospital Tianjin China

**Keywords:** antitumour, AZD2014, castration‐resistant prostate cancer, docetaxel, mTORC1 and 2

## Abstract

Recent studies indicate mammalian target of rapamycin (mTOR) may play an important role in PCa progression and drug resistance. Here, we investigated the effects of a novel mTORC1/C2 dual inhibitor, AZD2014, on naive and docetaxel (Doc)‐pre‐treated castration‐resistant PCa (CRPC) cells and explored its therapeutic potential in CRPCs. In the current study, AZD2014 has a greater inhibitory effect against 4EBP1 and AKT phosphorylation than rapamycin in CRPC cells and prevented the feedback activation of AKT signalling. Importantly, AZD2014 suppressed CRPC cell growth in vitro by suppressing proliferation, apoptosis, cell cycle arrest at G1 phase and autophagy to a greater extent than rapamycin. Moreover, AZD2014 was more efficacious than rapamycin in inhibiting migration, invasion and EMT progression in Doc‐sensitive and Doc‐resistant CRPC cells. Overall, AZD2014 showed significant antitumour effects. Thereby, the current study highlights a reliable theoretical basis for the clinical application of AZD2014 in both Doc‐sensitive and Doc‐resistant CRPCs.

## 1 INTRODUCTION

Prostate cancer (PCa) is one of the most commonly diagnosed malignancies, and the incidence of PCa is steadily increasing worldwide.^1^, ^2^ Most PCa patients who initially respond well to androgen deprivation therapy acquire resistance to this therapy and progress to a castration‐resistant prostate cancer (CRPC) state after a median time of 18‐24 months.^3^ Docetaxel (Doc), the current standard first‐line chemotherapy for metastatic CRPC, plays an important role in treating CRPC and can prolong overall survival and improve patient quality of life.^4^ After a period of Doc therapy, patients develop drug resistance and disease progression that can ultimately be life‐threatening.^4^ Among the available treatments for CRPC, the efficacy of conventional chemotherapy remains limited.^5^ Recently, cabazitaxel was designed to overcome Doc resistance in CRPC therapy, but cabazitaxel increased expression of the multidrug resistance 1 (MDR1) protein, induced anticancer drug efflux from the cell and caused a variety of serious side‐effect.^6^ Therefore, the need to find new methods to improve the efficacy of anticancer treatment in Doc‐resistant patients is urgent.

The phosphatidylinositol 3‐kinase (PI3K)/Akt/mammalian target of rapamycin (mTOR) pathway, one of the most commonly activated signalling pathways in many human cancers, is a key signalling pathway that controls cell growth, differentiation and metabolism.^7^ The PI3K/Akt/mTOR pathway was found to be inappropriately activated in PCa tissues by immunohistochemistry and was more prevalent in metastatic sites,^8^ as it was deregulated in 42% of localized disease cases and 100% of advanced‐stage disease cases.^9^ In addition to its role in regulating cell proliferation and invasion, the mTOR pathway is related to drug resistance.^10^, ^11^ Many investigations have shown that the PI3K/Akt/mTOR pathway is closely related to the progression of CRPC and the development of drug resistance.^12^ Therefore, targeting mTOR signalling is considered a very promising treatment for CRPC. In addition, the PI3K/Akt/mTOR pathway is a critical target for many other cancer treatments^13^, ^14^ mTOR exists in complexes called mTOR complex 1 (mTORC1) and mTOR complex 2 (mTORC2), which share subunits but have distinct cellular functions and localizations. The so‐called rapalogs, such as everolimus, an mTORC1 inhibitor, have been approved to treat certain types of cancer, such as kidney cancer and pancreatic neuroendocrine tumours.^15^, ^16^ Everolimus combined with Doc was shown to control the progression of disease in CRPC patients.^17^ However, androgens are vital factors that regulate tumour growth in CRPC patients and can activate the mTORC2 pathway, resulting in the activation of Akt and facilitating the survival of tumour cells. These results indicate a reciprocal feedback mechanism by which PI3K/Akt/mTOR signalling inhibits androgen receptor (AR) signalling, indicating a potential mechanism underlying the clinical inefficacy of mTOR inhibitors in CRPC.^18^ Preclinical studies investigating specific inhibitors of PI3K or mTOR yielded promising results; however, the evidence from clinical trials remains less convincing.

There are some dual mTORC1/2 inhibitors, such as AZD8055, OSI‐027, MTI‐31 (LXI‐15029), TAK‐228 and AZD2014. Many studies have confirmed that AZD2014 can inhibit the growth of pancreatic neuroendocrine tumours resistant to everolimus and that AZD2014 is also a highly effective treatment for renal cell carcinoma and ER^+^ breast cancer.^19^, ^20^, ^21^ Therefore, it is reasonable to hypothesize that simultaneously inhibiting mTORC1 and mTORC2 would have a greater antitumour effect than rapalogs or even reverse Doc resistance in these kinds of PCa cell lines. In this study, we revealed the potential impact of AZD2014 in impeding both mTORC1 and mTORC2 signalling in CRPC cells. Analyses of AZD2014 in cultured human PCa cell lines revealed that AZD2014 has broad effects on cancer resistance across both Doc‐sensitive and Doc‐resistant CRPC cell lines. These effects include antiproliferative effects and the ability of AZD2014 to induce apoptosis; inhibit migration, invasion and EMT progression; and activate autophagy. In addition, parallel studies showed that AZD2014 is more efficient than rapamycin and the mechanism of action of AZD2014 as an antitumour agent. These promising results provide rationale for the clinical assessment of AZD2014 in PCa therapy.

## 2 MATERIALS AND METHODS

### 2.1 Chemicals and antibodies

AZD2014, rapamycin and docetaxel were obtained from Selleck China. 3‐methyladenine (3‐MA) and monodansylcadaverine (MDC) stain (D4008) were obtained from Sigma‐Aldrich Corporation. Antibodies for anti‐light chain 3A/B (LC3A/B), Beclin‐1, p‐4EBP1 (T37/46), 4EBP1, p‐AKT (Ser‐473), total AKT, CDK4, cyclin D1, Bax, cleaved‐poly ADP‐ribose polymerase (PARP) (#5625), glyceraldehyde‐3‐phosphate dehydrogenase (GAPDH), β‐tubulin were purchased from Cell Signaling Technologies. Antibodies for E‐cadherin, N‐cadherin and Vimentin were obtained from Abcam.

### 2.2 Cell lines and cell culture

The docetaxel‐sensitive prostate cancer cell lines (C4‐2, CWR22RV1) were purchased from the American type culture collection (ATCC),US. Drug gradient generated docetaxel‐resistant prostate cancer cell lines (C4‐2 DOCR and CWR22RV1 DOCR, which were provided by Chawnshang Chang from University of Rochester, US). All the cell lines were grown in RPMI‐1640 (Gibco) containing 10% foetal bovine serum and incubated in a humidified atmosphere of 5% CO_2_ maintained at 37°C.

### 2.3 CCK8 cell proliferation assay

Cell proliferation was determined by using Cell Counting Kit‐8 (Beyotime Inst Biotech) according to the manufacturer's instructions. Briefly, 2000 cells/well were seeded in a 96‐well flat‐bottomed plate, and incubation with or without with AZD2014 or rapamycin, then grown at 37°C for several days. Cell growth was detected every 24 hours for 5 days. At each time‐point, every 100μl of Cell Counting Kit‐8 solution was dilution with 1 ml complete medium and incubated for 2 hours at 37°C. The absorbance was finally detected by a microplate reader at a wavelength of 450 nm (Bio‐Rad). Experiments were repeated at least three times.

### 2.4 Cell cycle

After 48 hours incubation with or without AZD2014 or rapamycin, cells were collected and fixed in 70% cold ethanol overnight at 4°C and then incubated with RNase A (Sigma) at 37°C for 30 min avoided from light and subsequently stained by propidium iodide (PI) (Nanjing KeyGen Biotech Co., Ltd.) staining solution. The samples were immediately were analysed on a FACSort flow cytometer (BD Biosciences). Data were processed by CELL quest software (BD Biosciences).

### 2.5 Flow cytometry assay

After 48h incubation with or without AZD2014 or rapamycin, cells were harvested and resuspended in fixation fluid. 5 µl Annexin V‐FIFC and 5µl propidium iodide were added to 500 µl cell suspension. Cell apoptosis was then determined by using FACSort flow cytometer (BD Bioscience). In the graphs, the quadrant, respectively, stands for dead cells, living cells, early apoptotic cells and late apoptotic cells.

### 2.6 Transwell assay

Cells were trypsinized and suspended again under RPMI 1640 medium including 1% FBS after incubation with or without AZD2014 or rapamycin for 48 hours, about 3 × 10^5^ cells with 200 μl serum‐free medium were plated into the upper chambers (24‐well insert, pore size 8 μm; Corning). For cell migration assays, in the lower chambers, 500 μl of RPMI 1640 with 20% FBS was added and then incubated at 37°C for 48 hours. Then, a cotton swab could be used to remove the cells on the top surface of the membranes, and the fixing and crystal violet colouring of membranes could be achieved within 20 minutes. And for cell invasion assays, the upper chambers were added with Matrigel (1:8.50 μl/well, BD Bioscience). Concurrently, the lower chambers were filled with 500 μl medium containing 10% foetal bovine serum. Cells were cultured at 37°C in a 5% CO_2_ atmosphere for 48 hours. Cells under the surface of the lower chamber were washed with 1 × PBS, fixed with 4% paraformaldehyde for 20 minutes, stained with 0.1% crystal violet for 25 min and then washed three times. The numbers of migrated or invaded cells were counted with an inverted microscope and imaged (Nikon). Experiments were repeated at least three times.

### 2.7 Wound‐healing assay

Cells motility was determined by wound‐healing assay. Cells were cultured in the presence or absence of AZD2014 or rapamycin for 48 hours and then spread within six well plate with 4 × 10^5^ cells density per well and cultivated into 90% confluent cells. Then, a wound field was created by using a sterile 200 μl pipette tip, which resulted in a denuded area with a fixed width. Phosphate buffered saline (PBS) was used to wash cells off cell debris, and culture medium was added to the cell culture. The cells were incubated for 48 hours at 37°C with or without AZD2014 or rapamycin, and then, the migration of cells was monitored with a digital camera system at different time‐points (0, 24 or 48 hours).The cell migration distance (μm) was calculated by the software programme HMIAS‐2000. Photographs were taken at 0 and 48 hours at the same position of the wound, and the distance between the edges was measured.

### 2.8 Immunofluorescence

Cells were cultured in a 24‐well plate and subjected to immunofluorescence analysis at 48 hours after incubation with or without AZD2014 or rapamycin. Briefly, cells were washed with cold PBS, immediately fixed using 4% paraformaldehyde in PBS for 15 minutes and permeabilized with 0.5% Triton X‐100 in PBS for 15 minutes. Cells were incubated with E‐cadherin, and vimentin antibody was used at a dilution of 1:100 overnight at 4^°^C. After washing in PBS, cells were incubated at 37^°^C for 1h with diluted FITC‐conjugated anti‐rabbit or antimouse secondary antibody. Last, cells were washed thrice with PBS and mounted with 4, 6‐diamidino‐2‐phenylindole, dihydrochloride (DAPI, Invitrogen Corporation, D1306) with concentration of 200 ng/ml on slides and sealed with transparent nail varnish. Images were photographed under a fluorescence microscopy (Olympus). Experiments were repeated at least three times.

### 2.9 Acidic vesicular organelles staining

To determine the presence of autophagic vesicles in prostate cancer cells, C4‐2, CWR22RV1, C4‐2 DOCR and CWR22RV1 DOCR cells were grown on cover slips inserted into a 6‐well plate and incubation with DMSO, 3MA, AZD2014, rapamycin or both of two for 48h. The formation of autophagosomes was specifically marked with monodansylcadaverine (MDC), following manufacturer's protocol. In brief, cells in each well were incubated with 100 μl of the MDC staining solution at 37°C for 20 minutes, which were protected from light and washed with 1 × PBS. Cell nuclei stained cyan‐green were positive for an acidic autophagosome. Finally, the cells were mounted on a glass slide and viewed under a fluorescence microscope (Olympus Corporation of the Americas, Waltham, MA, ix81), and we identified five microscope fields of every replication microscopic picture (×400) and the cells were counted. The results were presented by mean ± SD.

### 2.10 Western blot analysis

Cells were harvested at 48 hours following co‐cultured with AZD2014 or rapamycin at a certain concentration. Proteins were separated by 10% SDS/PAGE and transferred onto PVDF membranes (Millipore). After blocking with non‐fat dry milk for 1 hour at room temperature, the membranes were incubated overnight at 4°C with specific primary antibodies. After being washed three times in TBST, membranes were incubated with corresponding second antibody (1:5000) in blotting buffer for 60 min at room temperature and visualized by enhanced chemiluminescence (ECL) assay kit (Millipore). Western blot bands were exposed to X‐ray films and were quantified by Quantity One software. The relative expression of protein was standardized by comparison with anti‐GAPDH or anti‐β‐tubulin antibody. All antibodies used in this work were appropriately dilutions with 1:1000.

### 2.11 Statistical analyses

All experimental data from three independent experiments were analysed by Student's t test or chi‐square test, and results were expressed as mean ± SD. In all cases, *P*‐values <0.05 were considered to be statistically significant. All statistical tests were conducted by SPSS software (SPSS Standard version 19.0; SPSS Inc).

## 3 RESULTS

### 3.1 AZD2014 restrains both mTORC1 and mTORC2 signalling in CRPC cells

**Figure 1:**
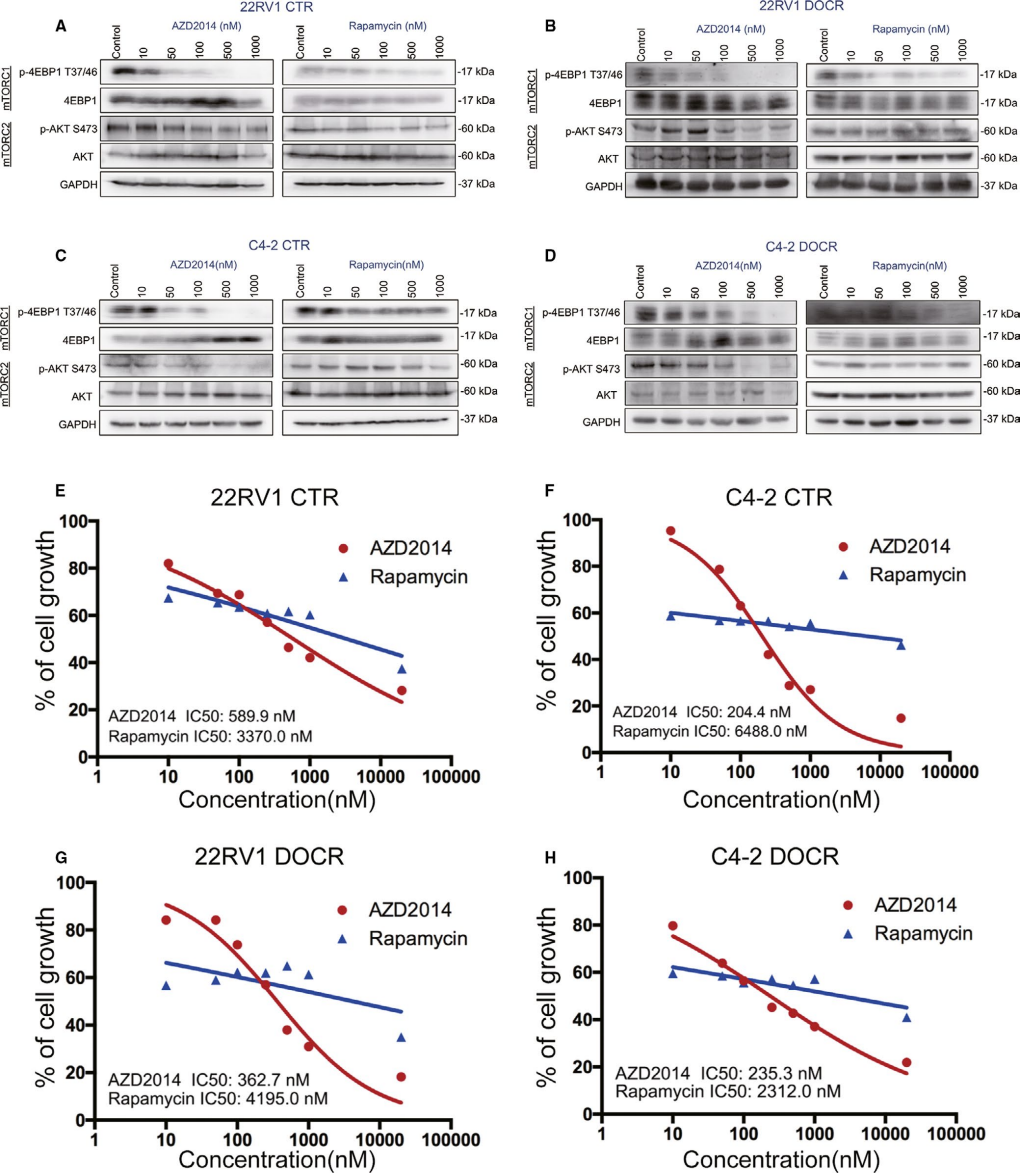
The biochemical activity of AZD2014 in blocking mTORC1/C2 signalling in prostate cancer cells. C4‐2, CWR22RV1, C4‐2 DOCR and CWR22RV1 DOCR cells were either treated with dimethyl sulfoxide (DMSO) (CTR) or increasing final concentrations of AZD2014 (10 nM, 50 nM, 100 nM, 500 nM, 1000 nM) or rapamycin (10 nM, 50 nM, 100 nM, 500 nM, 1000 nM) and further cultured for 48 hours before lysis and immunoblotting. A‐D, Expression of the mTORC1 and mTORC2 signalling proteins was tested by Western blotting. E‐H, IC50 values were generated by GraphPad Prism version 7.0 from CCK8 assay data. The experiments in this figure were repeated three times, which yielded similar results

AZD2014 is a dual inhibitor of mTORC1/2, and rapamycin is an mTORC1 inhibitor. We aimed to explore the biochemical activities of AZD2014 and rapamycin in controlling mTORC1 (p‐4EBP1) and mTORC2 (AKT phosphorylated at Ser473) signalling in CRPC cells. First, we detected the expression of 4EBP1 phosphorylated at Thr37/46 and AKT phosphorylated at Ser473 via Western blotting to test the mTOR signalling activation conditions in CRPC cells (the Doc‐sensitive C4‐2 and CWR22RV1 CRPC cell lines and the Doc‐resistant C4‐2 DOCR and CWR22RV1 DOCR CRPC cell lines, which were generated with a drug gradient) treated with AZD2014 and rapamycin at different concentrations. As shown by the results, in all the CRPC cell lines, AZD2014 effectively blocked the phosphorylation of 4EBP1 at Thr37/46 and that of AKT at Ser473 in a concentration‐dependent manner, and this inhibitory effect was especially significant when the concentration was 100 nM or more. In contrast, rapamycin also had a certain weak inhibitory effect on 4EBP1 phosphorylation at Thr37/46 in a concentration‐dependent manner but had little inhibitory effect on AKT phosphorylation at Ser473 even at a high concentration of 1000 nM (Figure 1A‐D). These results demonstrate that AZD2014 is a potent inhibitor of mTORC1 and mTORC2 in CRPC cells that blocks mTOR signalling more thoroughly than rapamycin. We performed a CCK8 assay to detect the half‐inhibitory concentration (IC50) of AZD2014 and rapamycin in four PCa cell lines and the effect of AZD2014 and rapamycin at their IC50 values on the proliferation of Doc‐sensitive/resistant PCa cells. The results indicated that AZD2014 and rapamycin dose‐dependently inhibited the survival of 22RV1, C4‐2, 22RV1 DOCR and C4‐2 DOCR cells (Figure 1E‐H) and that AZD2014 was significantly more effective than rapamycin at the same concentration (Figure 1E‐H). 22RV1, C4‐2, 22RV1 DOCR and C4‐2 DOCR cells were discovered to be sensitive to AZD2014, which exhibited IC50 values of 589.9, 204.4, 362.7 and 235.4 nM, respectively (Figure 1E‐H). However, rapamycin modestly decreased cell survival and exhibited IC50 values of 3370, 6488, 4195 and 2312 nM in 22RV1, C4‐2, 22RV1 DOCR and C4‐2 DOCR cells, respectively, which were higher than those of AZD2014 (Figure 1E‐H). In summary, we treated 22RV1, C4‐2, 22RV1 DOCR and C4‐2 DOCR cells with AZD2014 at concentrations of 600, 200, 360 and 240 nM, respectively, for all follow‐up experiments, and rapamycin at the appropriate dose was used as a control (CTR).

### 3.2 AZD2014 inhibits proliferation and induces apoptosis in CRPC cells

**Figure 2:**
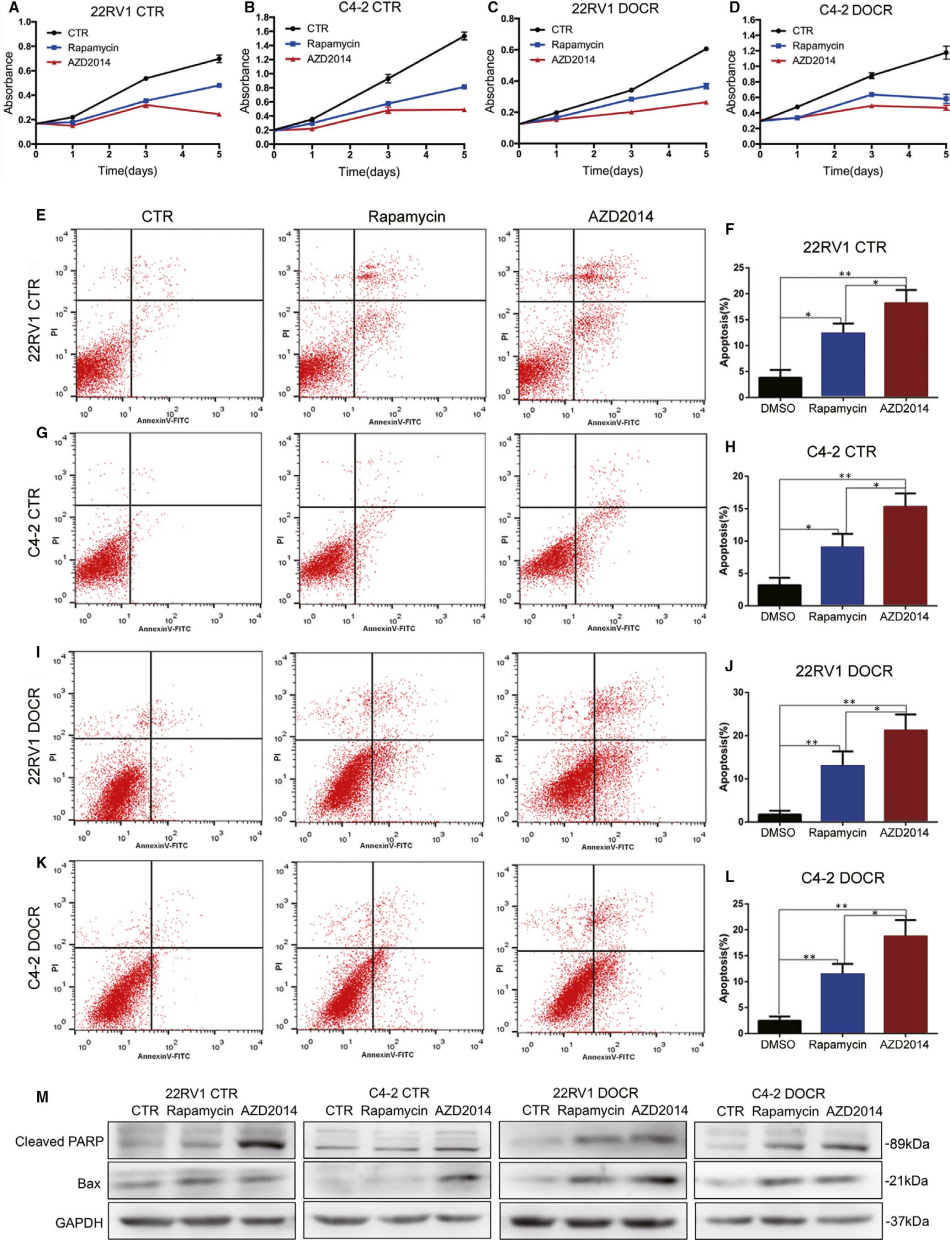
AZD2014 inhibited proliferation and induces apoptosis in CPRC cells. C4‐2, CWR22RV1, C4‐2 DOCR and CWR22RV1 DOCR cells were either treated with DMSO (CTR) or treated with the indicated concentrations of AZD2014 or rapamycin. The following experiments were performed after 48 hours. A‐D, Cell growth curves of all prostate cancer cell lines were determined by CCK8 assay every 24 hours. E, G, I, K, Cell apoptosis was detected by Annexin V fluorescence‐activated cell sorting (FACS), and the diagram shows representative apoptosis rates in prostate cancer cell lines treated with DMSO, AZD2014 or rapamycin. F,H,J,L, Statistical analysis of the apoptosis rates of prostate cancer cell lines. M, Western blot analysis of the relative expression of Bax and cleaved PARP. GAPDH served as a loading control. Data are presented as the mean ± SD from at least three independent experiments. (*P < .05, **P < .01)

To further explore the effects of AZD2014 and rapamycin on cell proliferation, we treated CRPC cell lines with AZD2014 at the appropriate concentration above for five days. Changes in the proliferation of PCa cells were measured with CCK8 assays. As shown in Figure 2A‐D, compared to the CTR group, cells in both tested groups displayed progressively retarded growth after 48 hours, and AZD2014 had a more significant antiproliferative effect than rapamycin on all four CRPC cell lines tested here. Then, we conducted a flow cytometry assay to detect the effects of AZD2014 and rapamycin on apoptosis in CRPC cells to examine whether cytotoxicity was associated with cell apoptosis. As shown in Figure 2E‐L, there was an increase in Annexin V/PI‐positive CRPC cells after treatment with AZD2014 and rapamycin. The percentage of apoptotic cells increased from 3.79 ± 0.89% in 22RV1 cells in the CTR group to 12.37% ± 1.09% (*P *< .05) and 18.24% ± 1.44% (*P *< .05) in 22RV1 cells treated with rapamycin and AZD2014, respectively (Figure 2E‐F); from 3.20% ± 0.66% in C4‐2 cells in the CTR group to 9.11% ± 1.16% (*P* < .05) and 15.34% ± 1.17% (*P* < .05) in C4‐2 cells treated with rapamycin and AZD2014, respectively (Figure 2G‐H); from 1.73 ± 0.52% in 22RV1 cells in the CTR group to 13.04% ± 1.93% (*P* < .05) and 21.20 ± 2.14% (*P* < .05) in 22RV1 DOCR cells treated with rapamycin and AZD2014, respectively (Figure 2I‐J); and from 2.47% ± 0.46% in C4‐2 DOCR cells in the CTR group to 11.44% ± 1.15% (*P* < .05) and 18.74% ± 1.82% (*P* < .05) in C4‐2 DOCR cells treated with rapamycin and AZD2014, respectively (Figure 2K‐L). We found that CRPC cell apoptosis was increased in both the early and late stages after treatment with AZD2014 and rapamycin, as detected by flow cytometry assays. Western blotting was further performed to detect the protein expression of cleavage poly ADP‐ribose polymerase (Cleaved PARP) and bax in CRPC cells treated with AZD2014 and rapamycin. The results demonstrated that the Cleaved PARP and bax proteins were expressed at higher levels in CRPC cells treated with AZD2014 and rapamycin (Figure 2M), and these results were similar to those of the flow cytometry assay. In summary, these results show that AZD2014 has a greater potential to induce apoptosis in CRPC cells than rapamycin.

At the same time, we also provide the similar dose of AZD2014 check in normal prostate epithelial cells (RWPE1, PWR‐1E) to detect whether the drug is specificity without affected the normal prostate epithelium. The CCK8 assay results showed that AZD2014 dose‐dependently inhibited the survival of RWPE1 and PWR‐1E cells which exhibited IC50 values of 3301 and 1986 nM, respectively (S 1A and C). AZD2014 had hardly inhibitory effect in RWPE1 and PWR‐1E cells at the final concentrations (200, 250, 400, 600 nM) in 48 hours (S 1B and D). All these results may indicate that AZD2014 show certain specificity in CRPC and minimal side‐effects in the normal prostate epithelium.

### 3.3 AZD2014 induces cell cycle arrest at G0/G1 phase in CRPC cells

**Figure 3:**
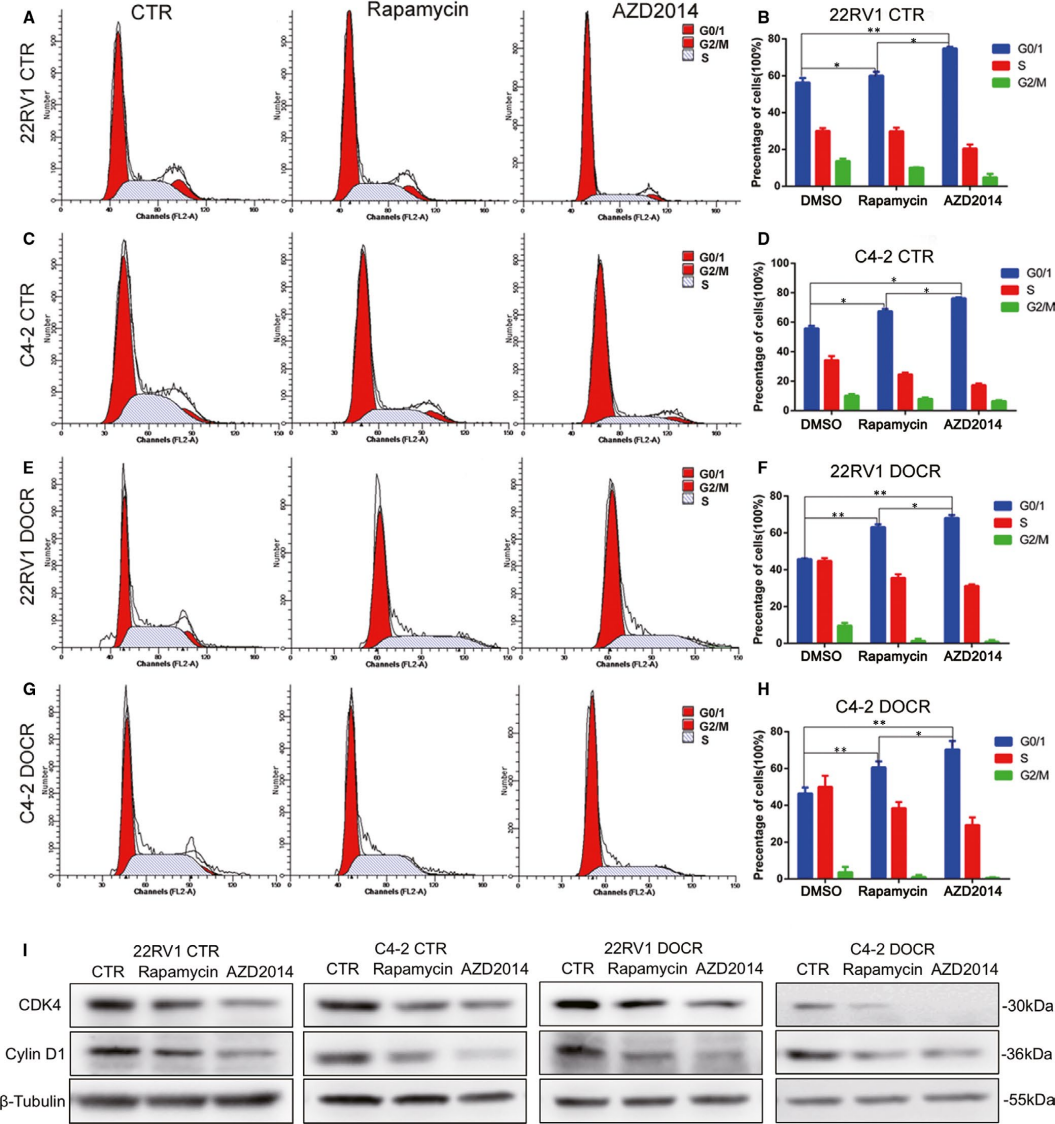
AZD2014 induced cell cycle arrest at G0/G1 phase in CRPC cells. C4‐2, CWR22RV1, C4‐2 DOCR and CWR22RV1 DOCR cells were treated with either DMSO (CTR) or AZD2014 or rapamycin at the indicated concentration and further cultured for several days. A‐H, After 48 hours, flow cytometry was used to determine the proportion of all prostate cancer cells in distinct cell cycle phases. I, After 48 hours, Western blot analysis of the relative expression of CDK4 and cyclin D1 was carried out. β‐Tubulin served as a loading control. The results are plotted as the mean ± SD of three independent experiments. (*P < .05,**P < .01; #*P* < .05, ##*P* < .01).

In the above experiments, we found that AZD2014 could significantly inhibit the proliferation of Doc‐sensitive and Doc‐resistant CRPC cell lines and had an obviously cytotoxic effect. Next, we aimed to understand whether the inhibitory effect of AZD2014 and rapamycin on proliferation was also related to the induction of cell cycle arrest. Many studies have shown that the mTOR signalling pathway is associated with cell cycle dysfunction,^22^ so we examined the effects of AZD2014 and rapamycin on the cell cycle in CRPC cell lines by flow cytometry analysis. Compared with cells in the CTR group, cells treated with AZD2014 and rapamycin showed a significant increase in the proportion of cells in G0/G1 phase (Figure 3A‐H). Furthermore, AZD2014‐treated CRPC cell lines showed a more obvious increase in G1‐phase cells than the corresponding cell lines treated with rapamycin (Figure 3A‐H), indicating that AZD2014 could induce robust G1 phase arrest in CRPC cell lines. Then, we detected expression of the cell cycle‐related proteins cyclin‐dependent kinase 4 (CDK4) and Cyclin D1, which regulate progression through the cell cycle and are particularly crucial during the transition from G1 to S phase. The protein expression of Cyclin D1 and CDK4 was reduced in AZD2014‐ and rapamycin‐treated CRPC cells compared with CTR cells (Figure 3I). Collectively, these results demonstrate that AZD2014 and rapamycin inhibit PCa cell proliferation through inducing cell cycle arrest at the G0/G1 phase in vitro.

### 3.4 AZD2014 activates autophagy in CRPC cells

**Figure 4:**
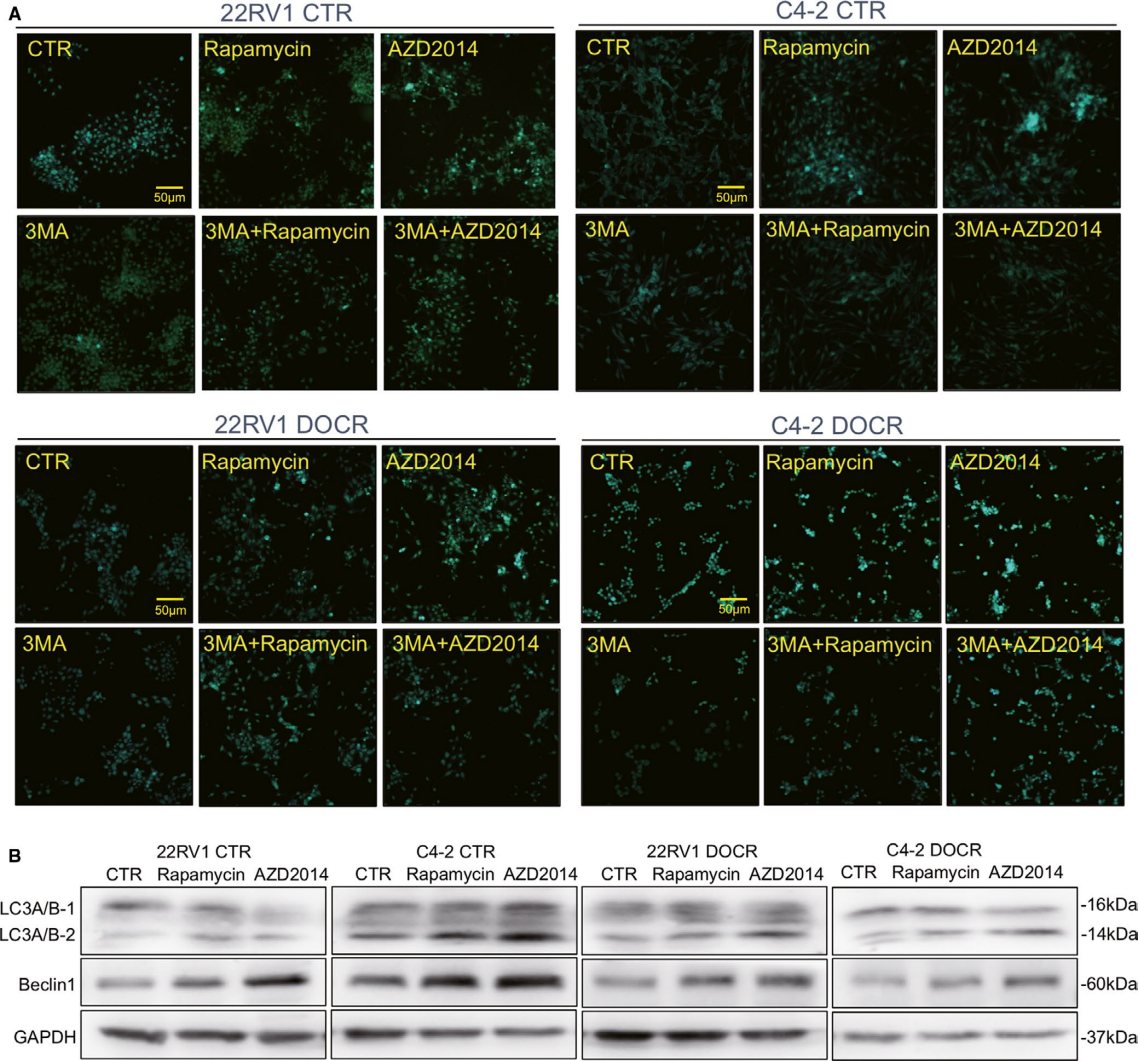
AZD2014 activated autophagy in CRPC cells. C4‐2, CWR22RV1, C4‐2 DOCR and CWR22RV1 DOCR cells were treated with either DMSO (CTR) or AZD2014 or rapamycin at the indicated concentration. The expression of related proteins was tested by Western blotting. A, Cells were treated with AZD2014 or rapamycin at concentrations of 200, 250, 400 and 600 nM for 48 hours in the presence or absence of 3‐MA. The cells were then stained with MDC to detect the formation of autophagosomes and immediately observed with a confocal microscope. Bars, 50 μm. B, The expression of Beclin‐1 and LC3A/B (LC3A/B‐1 and LC3A/B‐2) in prostate cancer cells treated as described above was assessed by immunoblot analysis

Many studies have shown that the inhibition of mTOR signalling is associated with the induction of autophagy.^23^ Therefore, we also evaluated whether AZD2014 affects this process. The CRPC cell lines were treated with AZD2014 and rapamycin, followed by monodansylcadaverine (MDC) staining to detect the formation of autophagic vacuoles. As shown in Figure 4A, both the number of MDC‐positive vacuoles and their size in CRPC cells were increased after treatment with AZD2014 and rapamycin. To further confirm this conclusion, we then treated CRPC cells with the autophagy inhibitor 3‐MA and determined whether the increase in the proportion of MDC‐positive cellular compartments was prevented. As speculated, 3‐MA inhibited the formation of MDC‐positive vacuoles induced by AZD2014 and rapamycin (Figure 4A), indicating that AZD2014 and rapamycin modestly activated the initiation of autophagy. Beclin‐1 and LC3A/B‐2, the most commonly monitored autophagy‐related proteins, play a critical role in the initiation of autophagy.^24^, ^25^ Western blotting results showed that AZD2014 and rapamycin activated autophagy in CRPC cells, as indicated by the significant up‐regulation of Beclin‐1 and an increase in the levels of LC3A/B‐2, consistent with the results of MDC staining (Figure 4B). In addition, AZD2014 treatment led to a more pronounced increase in the levels of LC3A/B‐2 than rapamycin (Figure 4B). These results suggest that the inhibition of mTORC1 by rapamycin had a less profound effect on the induction of autophagy than the blockade of mTORC1 and mTORC2 by AZD2014 in all four CRPC cell lines used here.

### 3.5 AZD2014 inhibits cell migration, invasion and EMT in CRPC cells

**Figure 5:**
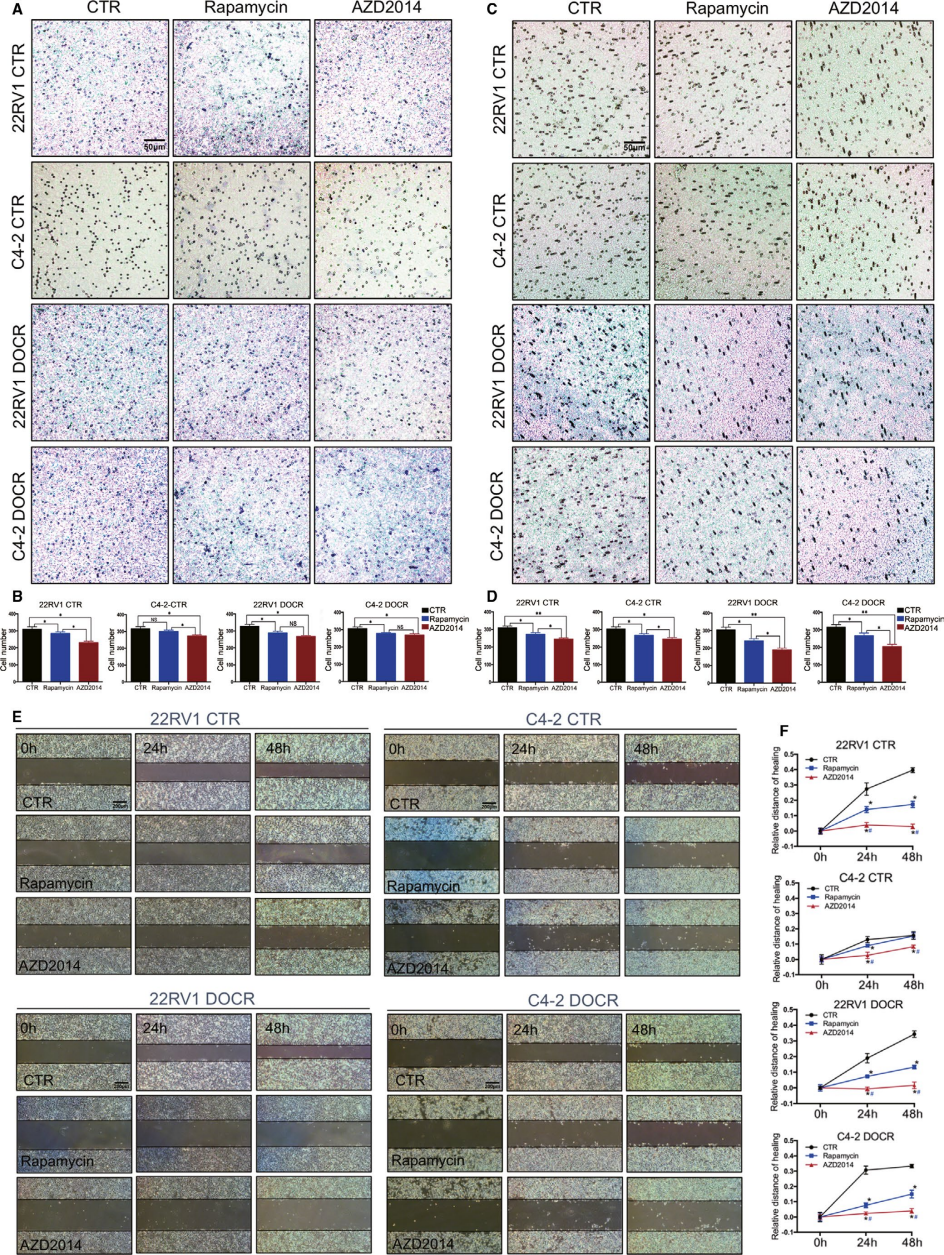
The effects of AZD2014 on migration and invasion in CRPC cells. C4‐2, CWR22RV1, C4‐2 DOCR and CWR22RV1 DOCR cells were treated with either DMSO (CTR) or AZD2014 or rapamycin at the indicated concentration. A, C, The invasive abilities of prostate cancer cell lines were determined with Transwell assays (representative photographs are magnified 200×). B, D, Representative quantification of migrated and invaded cells. E, The migratory abilities of prostate cancer cells were determined with wound‐healing assays (representative photographs are magnified 200×). F, Migrated cells were quantified. The inhibition of cell invasion by rapamycin and AZD2014 was observed in prostate cancer cells. The inhibition of cell migration by rapamycin or AZD2014 was observed in prostate cancer cells. Data are shown as the mean ± SD. (*P < .05, **P < .01)

**Figure 6:**
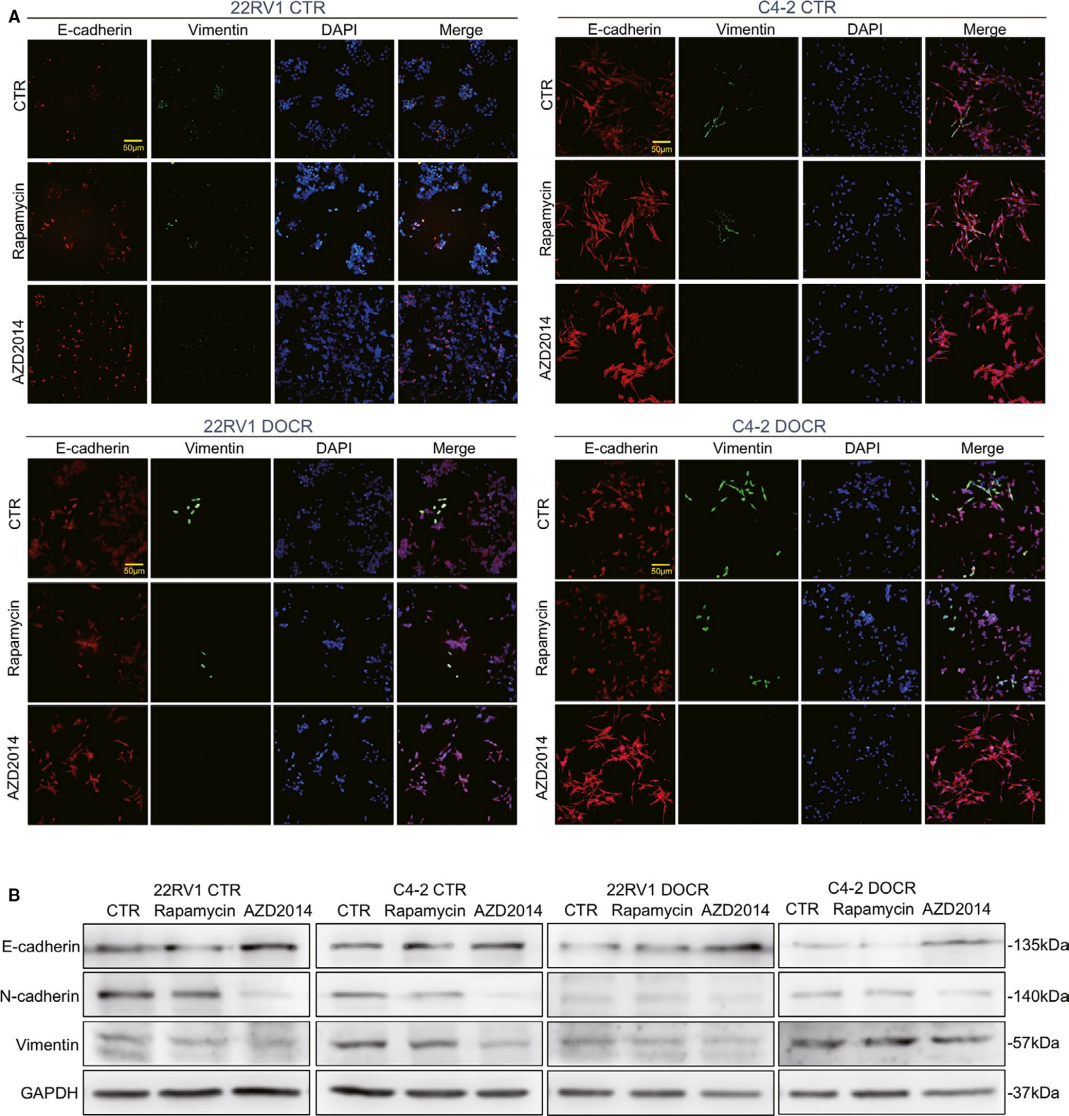
The effects of AZD2014 on EMT in CRPC cells. C4‐2, CWR22RV1, C4‐2 DOCR and CWR22RV1 DOCR cells were treated with either DMSO (CTR) or AZD2014 or rapamycin at the indicated concentration. The following experiments were performed after 48 hours. A, Immunofluorescence staining of prostate cancer cells treated as described above for E‐cadherin and vimentin. Bars, 50 μm. B, Prostate cancer cells were treated as described above, lysed and immunoblotted. The expression of EMT markers was detected via Western blotting. Rapamycin and AZD2014 increased the expression of E‐cadherin and decreased the expression of N‐cadherin and vimentin in prostate cancer cells. Data are shown as the mean ± SD. (*P < .05, **P < .01)

Recent studies have shown that inhibition of the mTOR signalling pathway significantly decreases the migration, invasion and EMT of PCa cells.^26^, ^27^ Therefore, we further determined whether AZD2014 regulates the migration, invasion and EMT progression of CRPC cells. The migratory abilities of CRPC cells were assessed by Transwell assays, which showed that treatment with AZD2014 and rapamycin notably decreased their migration (among 22RV1 cells, a mean of 229.6 and 284, respectively, vs 310 in the CTR group; among C4‐2 cells, a mean of 272 and 299, respectively, vs 316 in the CTR group; among 22RV1 DOCR cells, a mean of 267 and 287, respectively, vs 327 in the CTR group; among C4‐2 DOCR cells, a mean of 268 and 277, respectively, vs 292 in the CTR group) (Figure 5A‐B). The effect of AZD2014 on the invasive abilities of CRPC cells was determined using Matrigel invasion chamber assays. Decreased cell invasion was observed in all four CRPC cell lines treated with AZD2014 and rapamycin (among 22RV1 cells, a mean of 244 and 272, respectively, vs 309 in the CTR group; among C4‐2 cells, a mean of 244 and 266, respectively, vs 303 in the CTR group; among 22RV1 DOCR cells, a mean of 190 and 241, respectively, vs 304 in the CTR group; among C4‐2 DOCR cells, a mean of 204 and 266, respectively, vs 303 in the CTR group) (Figure 5C‐D). The effects of AZD2014 and rapamycin on CRPC cell invasion and migration were assessed in vitro, which showed that AZD2014 and rapamycin inhibited cell migration (Figure 5E‐F). In the process of EMT, the expression of intracellular epithelial markers (such as E‐cadherin) is down‐regulated, and some intracellular markers are up‐regulated (such as N‐cadherin and Vimentin)^28^ We then determined whether AZD2014 and rapamycin could regulate EMT in CRPC cells (Figure 6A). The expression of EMT markers was detected via immunofluorescence and Western blotting. The immunofluorescence results show that treatment of the CRPC cell lines with AZD2014 and rapamycin increased E‐cadherin expression but decreased N‐cadherin and vimentin expression. Western blotting confirmed the increased expression of E‐cadherin and decreased expression of Vimentin in CRPC cell lines treated with AZD2014 and rapamycin compared with the CTR groups (Figure 6B). These results indicated that AZD2014 is more effective than rapamycin in inhibiting cell invasion, migration and EMT in CRPC cells.

## 4 DISCUSSION

mTOR, a serine/threonine kinase, regulates cell growth and metabolism through two functionally distinct multiprotein complexes.^29^ mTORC1 mediates phosphorylation and activation of the p70S6 ribosomal kinase (S6K) and eukaryotic translation initiation factor 4E (eIF4E)‐binding protein (4E‐BP1), which together control protein synthesis.^30^ mTORC2 plays a role in cell growth and controls cell cycle‐dependent changes in the actin cytoskeleton.^31^ mTORC1 is affected by rapamycin treatment, while mTORC2 is thought to be rapamycin‐resistant, although prolonged treatment with rapamycin inhibits its assembly in some cell types.^32^ Some studies have indicated that mTORC2 is critical for the progression of PCa when PTEN is deleted in the prostate epithelium, while mTORC2 activity is not essential in the normal prostate epithelium,^33^ indicating that mTORC2‐specific inhibitors may be promising therapeutic agents for PCa. Similar results were confirmed in the treatment of colon cancer through the targeted inhibition of mTORC.^34^ Increasing preclinical evidence has shown the broad prospects of AZD2014 in antitumour therapy, and AZD2014 will soon enter clinical trials.^35^ However, there are still many important issues, such as genetic determinants, dosage and the time of drug use, that will ultimately determine its clinical success.^36^

Despite advances in the diagnosis and management of PCa, a large proportion of men progress to advanced or metastatic PCa, and morbidity from PCa remains high.^37^ At present, although androgen deprivation therapy through either surgical castration or chemotherapy remains the first‐line therapy for metastatic PCa, the effects of this treatment are temporary, and patients inevitably progress to CRPC.^38^ Berthold and his colleagues previously proposed chemotherapy consisting of Doc plus prednisone as the standard‐of‐care first‐line treatment for CRPC.^39^ Until recently, Doc‐based regimens offered a limited survival benefit with a high risk of the development of chemotherapy‐refractory disease. Several mechanisms have been proposed to interpret this resistance. Resistance to Doc appears to be mediated by tubulin mutation and the overexpression of multidrug‐resistant (MDR) genes, such as P‐glycoprotein (P‐gp) and β‐tubulin isotypes.^40^, ^41^ Much evidence has illustrated mTOR activation in numerous types of tumours, including PCa,^8^, ^34^ thus targeting mTOR has become a promising method for cancer therapy. However, the clinical results of the first‐generation mTOR inhibitor rapamycin and its derivatives in the treatment of most types of cancer have been disappointing. This may be due to the incomplete blockade of mTORC1, ineffective suppression of mTORC2 and feedback activation of AKT.^42^ These disappointing clinical results may also be due to mutations in tumour suppressor genes, and the abnormal expression of survival signalling factors involved in the PI3K/AKT/mTOR and/or MAPK/ERK signalling pathways.^43^, ^44^ These defects in clinical rapamycin‐based therapies have given rise to the development of novel mTOR inhibitors that can target the mTOR kinase domain and inhibit its catalytic activity via inhibiting both mTORC1 and mTORC2, thus preventing the feedback activation of Akt signalling.^45^

Our research confirmed that the new mTORC dual inhibitor AZD2014 can solve the above problems. In the current study, we clarified that AZD2014, a novel small‐molecular ATP‐competitive tyrosine kinase inhibitor (TKI) blocked a wide range of mTOR complex functions in both Doc‐resistant and Doc‐sensitive CRPC cell lines. Furthermore, the current study shows that AZD2014 had a more exhaustive inhibitory effect on mTORC1 than rapamycin and that the repression of mTORC2 prevented the activation of AKT signalling. mTOR signalling integrates multiple pathways involved in processes related to cell growth and proliferation, such as protein synthesis, cell cycle, apoptosis or autophagy, inside and outside the cell,^46^ so interference with mTOR signal transduction often affects cell proliferation and survival. As confirmed by our research, AZD2014 significantly induced apoptosis and G1 cell cycle arrest in Doc‐resistant CRPC cell lines by efficiently inhibiting mTORC1 and mTORC2 signals, which may be the main reason that AZD2014 inhibited CRPC cell proliferation. In addition, rapamycin could induce cell cycle arrest and partially inhibit proliferation by inhibiting mTORC1, suggesting that mTORC1 plays an important role in regulating the G1/S phase of the cell cycle. Another important function of the mTOR signalling pathway is its inhibition of autophagy.^47^ In fact, mTOR signalling is important for the induction of autophagy through the phosphorylation of autophagy‐related proteins.^48^ In our study, AZD2014 simultaneously inhibited both mTORC1 and TORC2, unlike rapamycin, which inhibited only mTORC1, suggesting further research on autophagy‐related mechanisms. In addition, the precise role of autophagy in tumour therapy remains controversial, so the autophagy‐related effects of AZD2014 in Doc‐resistant CRPC cells require further clarification. As increasing evidence supports the important roles of mTORC1 and mTORC2 in regulating cancer cell motility, EMT and metastasis, we also explored their related roles in this study. Our research confirmed that AZD2014 can significantly reduce the migration and invasion ability of Doc‐resistant CRPC cells, while the effect of rapamycin was relatively weak, indicating that both mTORC1 and mTORC2 are involved in regulating the migration and invasion capacity of CRPC cells. Previous studies have confirmed that disrupting the expression of mTORC1 or mTORC2 by RNA interference technology reduced the ability of a variety of cancer cells to migrate and invade.^49^, ^50^ A recent study showed that the inhibition of mTORC1 or mTORC2 reduced cell mobility by preventing rearrangement of the actin cytoskeleton and caused the formation of plate‐like pseudopods.^51^ During cell EMT, TGF‐β activates mTORC1 and mTORC2 signals through PI3K, resulting in increased protein synthesis, migration, invasion and epithelial‐mesenchymal phenotypic transformation.^43^ In this study, AZD2014, a dual inhibitor of mTORC1 and mTORC2, was more effective than rapamycin, which suppressed mTORC1 alone, in reversing the transformation of epithelial cells to mesenchymal cells. After CPRC cells were treated with AZD2014, the expression of E‐cadherin was increased, while N‐cadherin and vimentin expression was reduced. Thus, the concurrent inhibition of mTORC1 and mTORC2 may be a more effective strategy than the suppression of mTORC1 alone in PCa therapy and could especially be beneficial for patients who have become resistant to rapalogs.

The effects of AZD2014 on the CRPC cell lines in this study would be more compelling if they were further demonstrated in Doc‐sensitive and Doc‐resistant models in vivo. Currently, the mechanism of AZD2014 remains unclear; thus, further studies will provide assurance. Future exploration of predictive biomarkers of PCa susceptible to mTOR inhibitors may be a promising direction.

## 5 CONCLUSIONS

Our data demonstrated that AZD2014 was a highly potent inhibitor of both mTORC1 and mTORC2 and an effective antitumour agent for the treatment of CRPC in vitro, as treatment with AZD2014 led to the more profound inhibition of mTORC1 than rapamycin and inhibited mTORC2 without activating AKT signalling. AZD2014 participated in the regulation of various biological processes in PCa cells in vitro and was more effective than rapamycin. The underlying mechanism of the antitumour activity of AZD2014 is associated with its complete inhibition of both mTORC1 and mTORC2 substrates, and the inhibition of mTORC2 impedes AKT signalling feedback inhibition. These lines of evidence provide a molecular basis for the clinical application of a dual mTORC1/2 inhibitor and indicate a useful anticancer strategy for treating patients with both CRPC and Doc‐resistant CRPC.

## CONFLICT OF INTEREST

The authors declare that they have no competing financial interest.

## AUTHOR CONTRIBUTION

Senmao Li: Data curation (equal); Methodology (equal); Writing‐original draft (equal); Writing‐review & editing (equal). Jindong Sheng: Data curation (equal); Methodology (equal); Writing‐review & editing (equal). Zhenhua Liu: Methodology (supporting). Yu Fan: Supervision (supporting). Cuijian Zhang: Formal analysis (supporting). Tianjing Lv: Supervision (supporting). Shuai Hu: Supervision (supporting); Writing‐review & editing (supporting). Jie Jin: Data curation (lead); Funding acquisition (lead); Resources (lead); Supervision (lead); Writing‐review & editing (supporting). Wei Yu: Data curation (lead); Funding acquisition (lead); Resources (lead); Writing‐review & editing (lead). Yi Song: Project administration (lead); Resources (lead); Supervision (lead); Writing‐review & editing (lead).

## Supporting information

Supplementary Material

## Data Availability

The data that support the findings of this study are available from the corresponding author upon reasonable request.
