# Supplementary material for: Potent antitumour of the mTORC1/2 dual inhibitor AZD2014 in docetaxel‐sensitive and docetaxel‐resistant castration‐resistant prostate cancer cells
Source: J Cell Mol Med. 2021 Jan 28;25(5):2436–49. doi: 10.1111/jcmm.16155 (PMC7933970; doi:10.1111/jcmm.16155)
Supplement: Supplementary file 1 — Supplementary Material [file JCMM-25-2436-s001.docx]

**Supplementary materials**

**Supplementary 1**

**
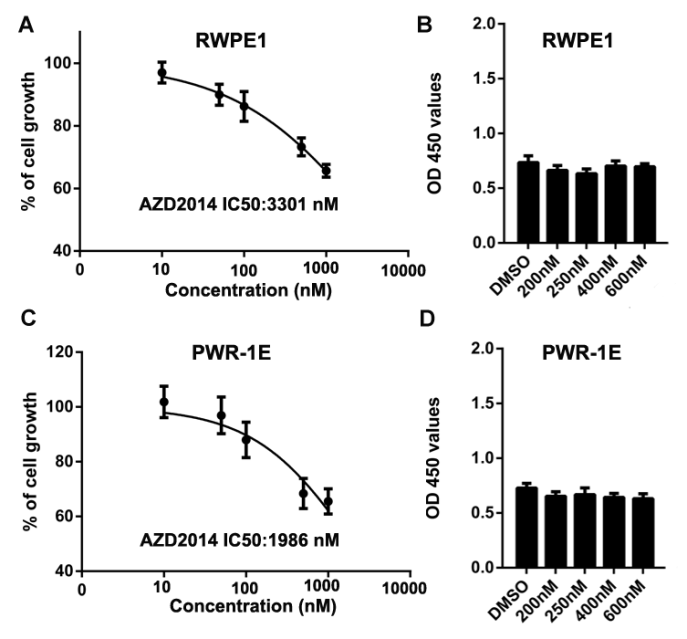
**

**S1. The proliferative effect of AZD2014 in normal prostate epithelial cells.**

RWPE1 and PWR-1E cells were either treated with dimethyl sulfoxide (DMSO) or increasing final concentrations of AZD2014 (10 nM, 50 nM, 100 nM, 500 nM, 1000 nM) or (200 nM, 250 nM, 400 nM, 600 nM) and further cultured for 48 hours. **A and C:** IC50 values were generated by GraphPad Prism version 7.0 from CCK8 assay data in 48 hours. **B and D:** Cell growth curves of both normal prostate epithelial cell lines were determined by CCK8 assay in 48 hours and OD values represented cell proliferation. The experiments in this figure were repeated three times, which yielded similar results.
